# Endoscopic treatment of nonoperable large postsurgical esophageal fistulas: retrospective analysis of a single tertiary center cohort

**DOI:** 10.1016/j.igie.2024.03.001

**Published:** 2024-03-26

**Authors:** Sebastian Petruzzella, Elodie Romailler, Thomas Greuter, Sarra Oumrani, Domenico Galasso, Maxime Robert, Styliani Mantziari, Markus Schäfer, Sébastien Godat

**Affiliations:** 1 Faculty of Biology and Medicine, University of Lausanne, Lausanne, Switzerland; 2 Division of Gastroenterology and Hepatology, Centre Hospitalier Universitaire Vaudois, Lausanne, Switzerland; 3 Division of Gastroenterology, Hôpital Riviera-Chablais VD-VS, Rennaz, Switzerland; 4 Division of Visceral Surgery, Centre Hospitalier Universitaire Vaudois, Lausanne, Switzerland

## Abstract

**Background and Aims:**

Anastomotic fistulas are a frequent and dreaded adverse event of esophagectomy. Endoscopic therapy using different techniques is now a well-established first-line treatment option. The aim of our study was to evaluate the efficacy of such endoscopic treatments in patients not fit for surgical reintervention and particularly in cases of major tissue defects of >10 mm.

**Methods:**

Fifty-seven patients with postoperative large esophageal fistulas who were not considered for surgical reintervention were retrospectively analyzed after undergoing treatment with different endoscopic techniques in a single tertiary center. The primary endpoint was to evaluate the technical and clinical efficacy of endoscopic treatments of those fistulas. The secondary endpoint was to evaluate the endoscopic treatment–related adverse events.

**Results:**

In 94.7% of patients (n = 54), the intervention was effectively carried out from a technical point of view. In 77.2% of patients (n = 44), treatment led to successful complete closure of the fistula. If we consider the 54 patients in whom technical success was reached, in 75.9% of them (n = 41), clinical success with complete closure of the fistula was achieved. Minor adverse events related to the procedure occurred in 26.32% of patients (n = 15) and major adverse events in 8.8% (n = 5). The mortality rate related to the procedure was 3.5% (n = 2).

**Conclusions:**

Endoscopic treatment is a technically achievable, highly effective way of treating postoperative large esophageal fistulas in patients who were not considered fit for surgical treatment, including major defects of >10 mm. It allows patients with a high risk of rapid deterioration to safely recover from their condition, avoiding severe and fatal adverse events without having to resort to debilitating surgical treatment.

Adenocarcinoma and squamous cell carcinoma represent more than 95% of esophageal cancers.^1^ Squamous cell carcinoma is the most prevalent worldwide, and its most notable risk factors are smoking and alcohol consumption. Adenocarcinoma, on the other hand, is most common in developed countries and is strongly related to GERD and Barrett’s esophagus.^2^ Most commonly, surgical resection, with or without neoadjuvant therapy, is the treatment of choice for advanced esophageal cancers. Surgical approaches include among others the Ivor-Lewis and McKeown techniques. For early-stage tumors, minimally invasive procedures like endoscopic submucosal dissection have been proven to provide a better technical and clinical outcome and are now preferred to open esophagectomy.^3^ Esophageal surgery can also be performed in other contexts, such as the treatment of gastroesophageal junction neoplasia or Boerhaave syndrome.

Postoperative adverse events may occur in up to 60% of patients, among which 13% are anastomotic leaks.^4^ Leaks have a significant morbidity and mortality rate, and urgent care is mandatory.^5^^,^^6^ The diagnosis can be established by imaging or EGD after clinical suspicion. Endoscopic treatment of anastomotic leaks is currently a well-established treatment option that avoids surgical management.^7^ The most common endoluminal approaches include self-expandable metal stents (SEMSs), clips, and vacuum-assisted closure (VAC) therapy.^8^ In this study, we report outcomes of endoscopic treatment in the management of anastomotic leaks after esophageal surgery in patients who were not considered fit for surgical reintervention and in cases of major defect tissue defined as fistulas >10 mm.

## Methods

### Study design

This was a retrospective single-center study conducted in the Department of Gastroenterology and Hepatology at the University Hospital in Lausanne, Switzerland. All endoscopic procedures for the treatment of postoperative esophageal anastomotic fistulas performed between January 2015 and August 2021 were analyzed. The study was approved by the local ethical board committee (CER-VD ID: 2022-00295).

### Patients

Patients aged ≥18 years who underwent primary endoscopic treatment for a postoperative esophageal anastomotic fistula measuring >10 mm were included. Exclusion criteria were age <18 years and refusal to be included in the study. Patients who could not be followed were also excluded. Age, sex, smoking status, at-risk alcohol consumption, steroid use, and proton pump inhibitor use before the procedure were retrospectively obtained from the patients’ medical reports. Neoadjuvant chemotherapy and radiotherapy treatments before surgery were also documented as well as the indication for surgery, histologic characterization of neoplastic lesions, surgery technique, and resection margin status.

Esophageal fistulas were either diagnosed by imaging or EGD after clinical suspicion and were characterized during the endoscopic procedures by their distance from incisors, length, and diameter with major defect defined as superior to 10 mm. The presence of sepsis at the time of anastomotic fistula diagnosis was documented.

Patients were treated with differently sized SEMSs, through-the-scope clips, over-the-scope clips (type GC; Ovesco, Tübingen, Germany), and VAC therapy.^8^ Bigger fistulas with large cavities and fluid collections at the initial presentation were treated with VAC covered by SEMSs, whereas smaller fistulas were managed with SEMSs only. As the treatment progressed, through-the-scope clips and over-the-scope clips were used to close smaller defects. Treatment was conducted until the fistula was successfully closed or failure, defined as death of the patient or the need for emergent surgery. The number of interventions needed were counted, and the time taken to close the fistulas was recorded.

### Endpoints

The primary endpoints of this study were technical and clinical efficacy of the endoscopic treatment of esophageal anastomotic fistulas in patients not fit for surgical treatment including patients with major tissue defect as previously defined. Technical efficacy was defined as a successfully completed procedure as initially planned, and clinical efficacy was defined as complete closure of the fistula at the end of endoscopic treatment, with patients discharged from acute care and tolerating oral intake. Prolonged treatment and a large number of endoscopic procedures were not defined as clinical failure, because surgical management options were not considered. Secondary outcomes were minor adverse events (grades I-II according to the Clavien-Dindo classification), defined as pain or fever after the endoscopic intervention, and major adverse events (grades III-V according to the Clavien-Dindo classification), defined as massive hemorrhage needing reintervention and collections needing either endoscopic, interventional radiology, or surgical treatment.

### Follow-up

We stopped follow-up in mid-2021 to have at least 6 months of follow-up after the end of endoscopic treatments of the last patient included.

### Statistical analysis

The statistical package program STATA (version 18; StataCorp, College Station, Tex, USA) was used. Metric data are shown as mean ± standard deviation (for normal distribution) or median (interquartile range [IQR]) (for non-normal distribution). Categorical data are summarized as the percentage of the group total. Categorical data were compared using the χ_2_ test and numerical data with the Student *t* test. To calculate the time taken to close the fistula, Kaplan-Meier curves were computed. A 2-sided *P* < .05 was regarded as statistically significant.

## Results

### Patient characteristics

We included 16 women (28.1%) and 41 men (71.9%) with a mean age of 63 ± 10 years (Table 1). Thirty patients were active or former smokers (52.6%), with a consumption ranging from 20 to 100 pack-year. Ten patients (17.5%) had an at-risk alcohol consumption, 24 patients (42.1%) used proton pump inhibitors, and 2 patients (3.5%) used steroids before the procedure. Forty-six patients (80.7%) received neoadjuvant chemotherapy and 23 patients (40.4%) received neoadjuvant radiochemotherapy, including 17 (29.8%) who had a 41-Gy protocol and 6 (10.6%) a 50-Gy protocol.

**Table 1:** Characteristics of patients and procedures

Characteristics	Values
Patients	
Sex	
Female	16 (28.1)
Male	41 (71.9)
Age at diagnosis, y	62.9 ± 10.2
Smoking	
Yes	30 (52.6)
No	27 (47.4)
At-risk alcohol use	
Yes	10 (17.5)
No	47 (82.5)
Steroid use	
Yes	2 (3.5)
No	55 (96.5)
Proton pump inhibitor use before surgery	
Yes	24 (42.1)
No	33 (57.9)
Preoperative radiotherapy	
Yes	23 (40.4)
41-Gy protocol	17 (29.8)
50-Gy protocol	6 (10.6)
No	34 (59.6)
Preoperative chemotherapy	
Yes	46 (80.7)
No	11 (19.3)
Procedures	
Indication for surgery	
Squamous cell carcinoma	18 (31.6)
Adenocarcinoma	29 (50.9)
Gastric cancer	4 (7.0)
Other cancer	4 (7.0)
No cancer	2 (3.5)
Surgical technique	
Ivor-Lewis	39 (68.4)
McKeown	7 (12.3)
Laparoscopic total gastrectomy with Roux-en-Y bypass	5 (8.8)
Other total gastrectomy	4 (7.0)
Other	2 (3.5)
Resection margins	
R0	41 (71.9)
R1	9 (15.8)
R2	1 (1.8)
Not determined	6 (10.5)

Values are n (%) or mean ± standard deviation.

Histologically, 18 lesions (31.6%) were squamous cell carcinomas, 29 (50.9%) adenocarcinomas, 8 (14%) other cancerous lesions, and 2 (3.5%) were non-neoplastic lesions. An Ivor-Lewis esophagectomy was performed on 39 patients (68.4%), a McKeown esophagectomy on 7 patients (12.3%), and a laparoscopic total gastrectomy with a Roux-en-Y reconstruction on 5 patients (8.8%). Resection margins were classified as R0 for 41 patients (71.9%), R1 for 9 patients (15.8%), R2 for 1 patient (1.8%), and could not be determined for 6 patients (10.5%). Median length of hospitalization was 52 days (IQR, 37-69).

### Fistula characteristics

Fistulas were diagnosed either with a CT in 17 patients (29.8%) or endoscopically in 40 patients (70.2%). The mean distance from the incisor to the fistula was 27.0 ± 7.3 cm (Table 2). In 25 patients (43.9%) the fistula measured 10 to 20 mm and in 32 (56.1%) >20 mm. Twenty-one patients (36.8%) had a fistula that measured >50% of the esophageal circumference in length. Median time from surgery to the diagnosis of fistula was 8 days (IQR, 4-13), and 21 patients (36.8%) were diagnosed with sepsis in the meantime.

**Table 2:** Characteristics of fistulas

Characteristics	Values
Average height of fistula from dental arches, cm	27.0 ± 7.3
Fistula size	
10-20 mm	25 (43.9)
>20 mm	32 (56.1)
Fistula longer than .5 times the diameter of esophagus	
Yes	21 (36.8)
No	36 (63.2)
Type of diagnosis	
Radiological examination	17 (29.8)
Endoscopic examination	40 (70.2)
Sepsis at time of diagnosis	
Yes	21 (36.8)
No	36 (63.2)
Time from surgery to diagnosis of fistula, days	8 (4-13)
No. hospitalized in the intensive care unit	37 (64.9)
No. hospitalized in the intermediate care unit	8 (14.0)

Values are n (%), mean ± standard deviation, or median (interquartile range).

### Endoscopic intervention characteristics

The median time from the diagnosis of the fistula to the beginning of endoscopic treatment was 0 days (IQR, 0-1), and the median time from the surgical procedure to the first endoscopic procedure was 10 days (IQR, 5-16) (Table 3). A median of 4 total endoscopic sessions (IQR, 3-7) was performed. The median time from the first intervention to successful closure of the fistula was 45 days (IQR, 29-83), and the median observation time, which also included patients for which fistula closure could not be achieved, was 48 days (IQR, 30-92).

**Table 3:** Characteristics of endoscopic intervention

Characteristics	Values
Time from fistula diagnosis to endoscopic treatment, days	0 (0-1)
Time from surgery to endoscopic treatment, days	10 (5-16)
No. of endoscopic sessions	4 (3-7)
Duration of treatment, days	45 (29-83)
Observation time, days	48 (30-92)
Time from intervention to closure of fistula, days	52 (40-99)
Duration of hospitalization, days	45 (29-83)
Fistula closed	
Yes	44 (77.2)
No	13 (22.8)
Technical success	
Yes	54 (94.7)
No	3 (5.3)
Technique used	
SEMS	29 (50.9)
SEMS + clip	11 (19.3)
SEMS + VAC	11 (19.3)
SEMS + VAC + clip	5 (8.8)
VAC + clip	1 (1.8)
Fistula adverse events	
Patient reoperated	12 (21.1)
Endoscopic treatment adverse events	
Minor adverse events	15 (26.3)
Major adverse events	5 (8.8)
Patient died	2 (3.5)

Values are median (interquartile range) or n (%).

*SEMS*, Self-expandable metal stent; *VAC*, vacuum-assisted closure.

Regarding the technique and material used, 29 patients (50.9%) were treated with 1 or repeated applications of a SEMS, 11 (19.3%) were treated with a combination of 1 or repeated applications of a SEMS and clips, 11 (19.3%) with a combination of repeated applications of a SEMS and VAC, 1 (1.8%) with a combination of repeated applications of VAC and clips, and 5 (8.8%) with a combination of repeated applications of SEMSs, VAC, and clips. Of these 57 patients, 37 (64.9%) were hospitalized in the intensive care and 8 (14.0%) in the intermediate care unit.

### Endpoints

After endoscopic treatment, technical success was carried out in 54 patients (94.7%). Clinical success was achieved in 44 patients (77.2%), and in all the clinical success cases, technical success was also achieved. No significant difference in clinical success was observed in groups having a different radiochemotherapy status and fistulas measuring more than half the circumference of the esophagus (*P* > .1). The average anastomosis height was not significantly different in the clinical success and failure groups (*P* > .1).

Minor adverse events (Clavien-Dindo grades I-II) after endoscopic treatment were reported in 15 patients (26.3%). Major adverse events (Clavien-Dindo grades III-V) were reported in 5 patients (8.8%).

**Figure 1:**
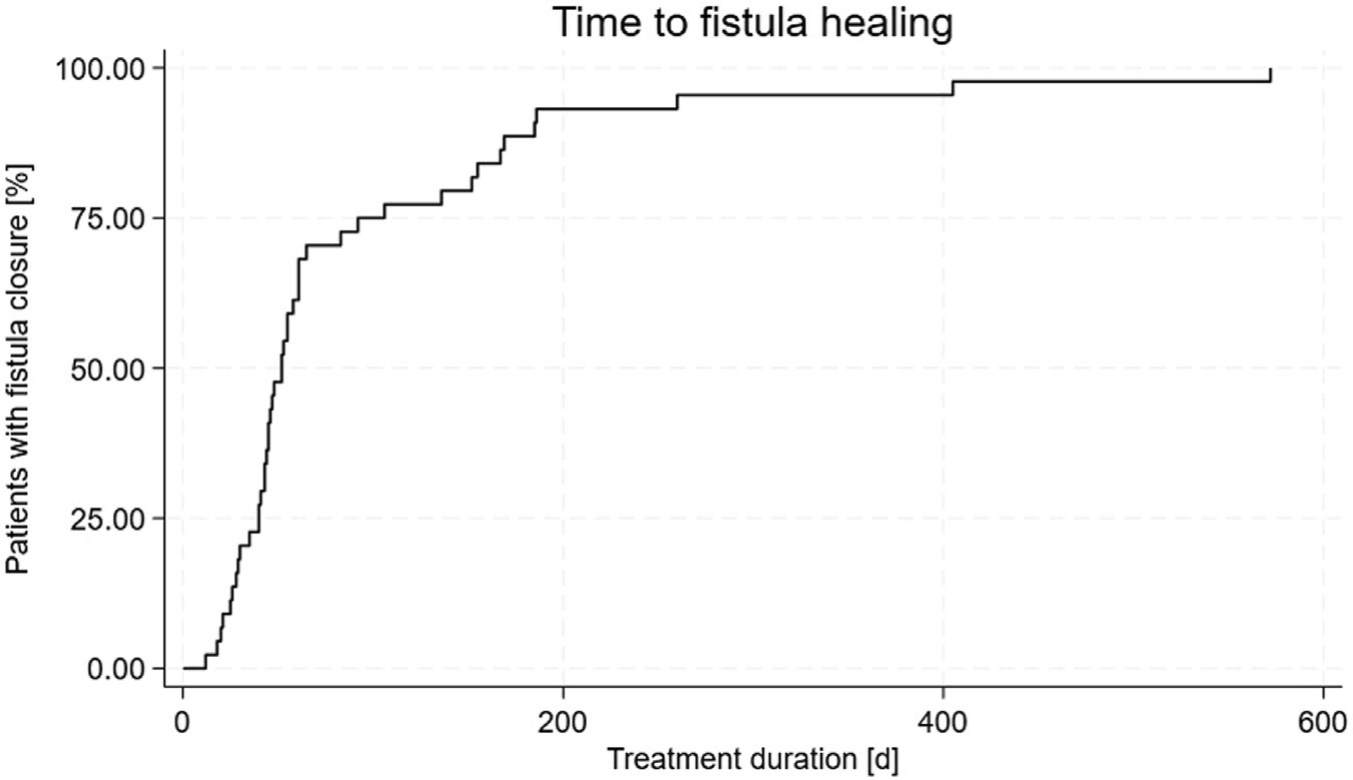
Time to fistula healing.

**Figure 2:**
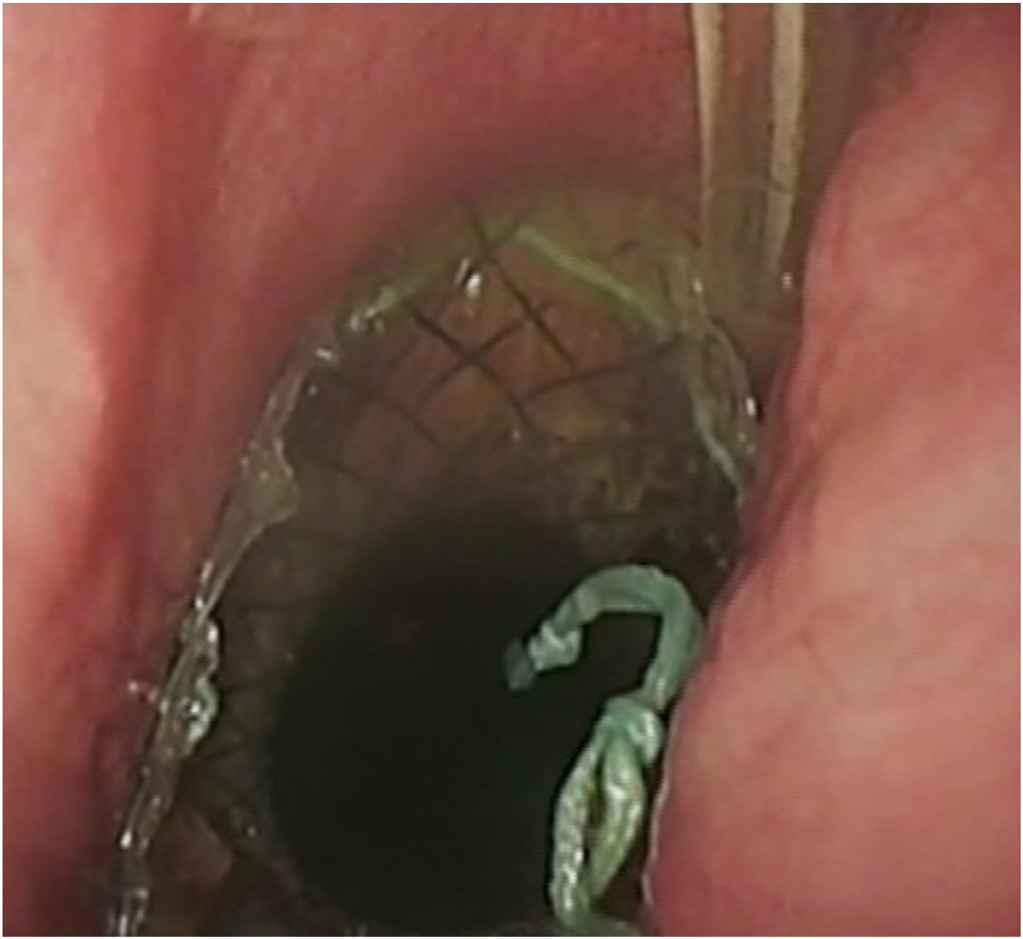
An example of a combined treatment with vacuum-assisted closure and self-expandable metal stent.

**Figure 3:**
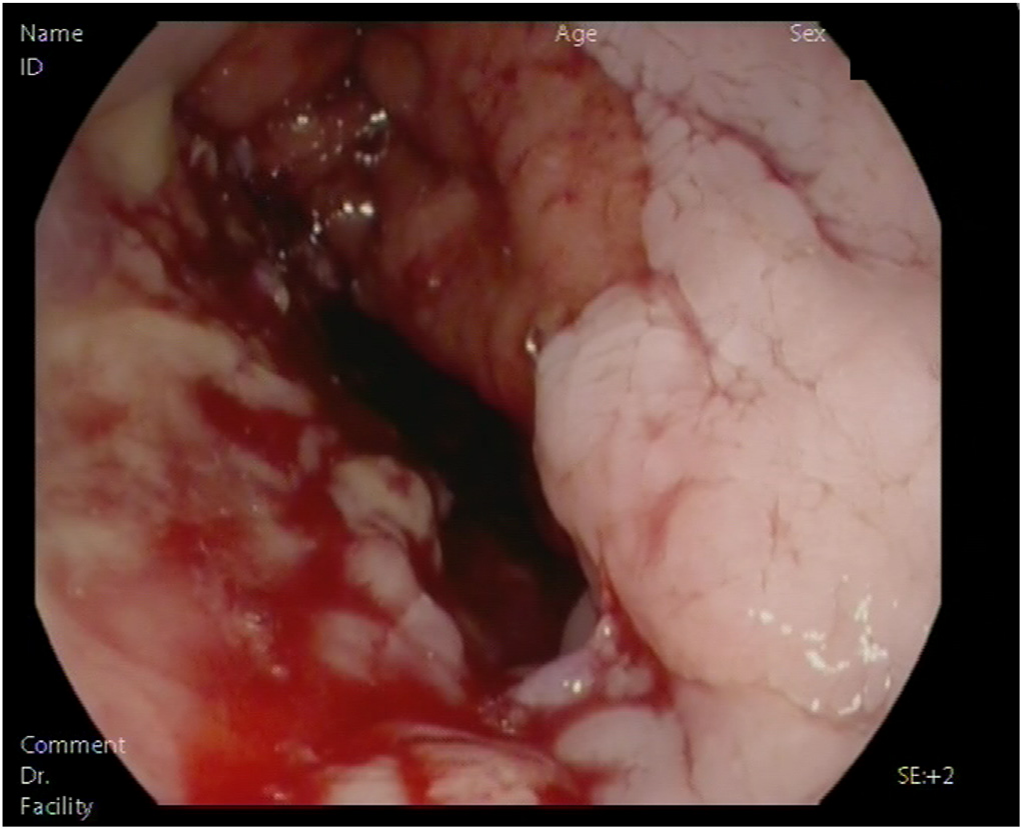
Esophageal fistula.

**Figure 4:**
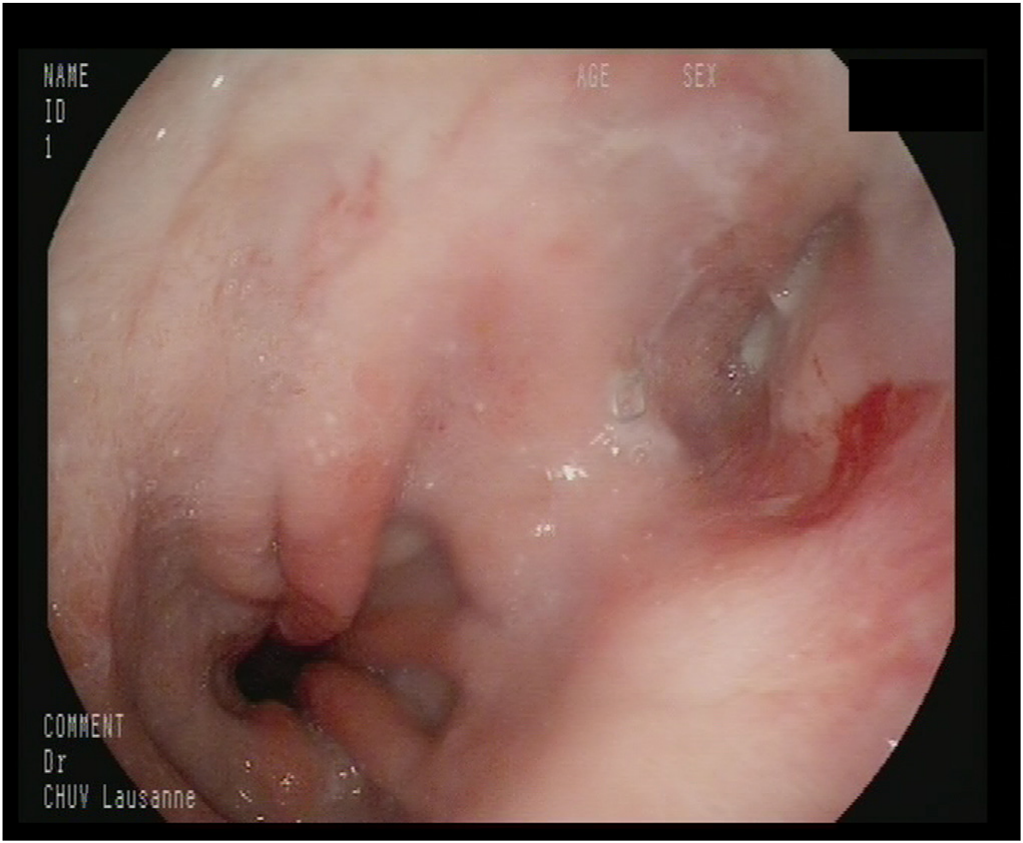
Healed fistula.

The mortality rate of the patients who had major adverse events related to the procedure was 3.5% (n = 2). Both patients had an upper GI bleed that could not be surgically or endoscopically treated (Figure 1, Figure 2, Figure 3, Figure 4).

## Discussion

Worldwide, esophageal surgery is complicated by anastomotic leaks in 13% of patients, and endoscopy is a well-established treatment option, including the use of SEMSs, clips, and VAC therapy. Although endoscopic options to treat esophageal fistulas were introduced in the early 2000s,^9^ only a few studies considering the efficacy and related adverse events of endoscopic treatment of surgical anastomotic fistulas are available, each including about 20 to 35 participants.^10^^,^^11^ Furthermore, as described in Dasari et al,^10^ many available studies are not based on anastomotic fistulas exclusively but include esophageal perforations as well. Our study included 57 patients with only postoperative esophageal anastomotic fistula, which to our knowledge is one of the largest retrospective studies on this topic. Moreover, all fistulas in our cohort measured >10 mm and were therefore considered as large. We found a technical success of endoscopic therapy of 94.7% and a clinical success of 77.2%. If we consider only patients who reached technical success, clinical success was 75.9%. The results available in the current literature report technical success rates of around 90% and clinical success of around 80% using SEMSs^10^ and similar outcomes in VAC treatment performed in patients refractory to stent placement,^8^ which is comparable with our results.

Minor adverse events according to the Clavien-Dindo classification as grades I to II were observed in 26.3% of patients. Major adverse events according to the Clavien-Dindo classification as grades III to V occurred in 8.8% of patients. One patient had septic shock because the fistula’s orifice was uncovered by the previous stent; he was treated by a new endoscopic procedure with a new SEMS. One patient had a new anastomotic leak with a tracheoesophageal fistula after a previous successful endoscopic closure, and he was finally treated by surgery. One patient had a SEMS migration with a jejunal perforation and was treated by surgery. Two patients (3.5%) had a massive hematemesis and cardiopulmonary arrest after the SEMS placement and died after the procedure.

Actual guidelines strongly recommend stent placement and other endoscopic techniques to treat anastomotic fistulas, whereas surgical management is reserved for early fistulas arising from technical failure or fistulas that failed endoscopic therapy.^12^ Nevertheless, the most recent European Society of Gastrointestinal Endoscopy guidelines acknowledge that the evidence lacks in quality.^7^ Endoscopic treatment of fistulas ≥1 cm led to fistula closure in 77.2% of cases in our series. Our data showed how endoscopic treatment has a technical failure rate of 5% and a major adverse event (Clavien-Dindo grades III-V) rate of 8.8%, which proved the feasibility and safety of this kind of endoluminal treatment in fragile patients who are not surgical candidates. Although relatively safe and effective, endoscopic treatment for esophageal fistulas has been shown to take a long time, with a median of 52 days from intervention to closure of the fistula, and to be resource expensive, with a median of 4 endoscopic sessions and most patients hospitalized in either the intensive care unit or an intermediate care unit. Such clinical commitment must be put in context, considering that all previous lines of treatment were not considered in this group of patients. Many major adverse events could be successfully managed. In 2 of 5 patients, the anastomosis was surgically reconstructed, and in 1 patient a hematoma was surgically evacuated, and endoscopic treatment succeeded thereafter. Reported mortality rates of postoperative fistulas can be as high as 35%.^5^ In our study, only 2 deaths could be linked to major adverse events of the treatment, albeit not as the only direct cause.

The retrospective, single-center design of this study has its limitations. A limited number of operators performed the procedures, and given the lack of detailed guidelines, the choice of technique was mainly dictated by the operators’ experience. The data sampled from already existing databases limited a more accurate characterization of the procedures and fistulas.

Future perspectives randomized controlled trials and larger well-designed cohort studies are required to improve the actual recommendations. A randomized controlled trial comparing interventional endoscopy, conservative treatment, and surgical reintervention could lead to a more transparent understanding of the need and efficacy of endoscopic treatment for esophageal fistulas, but considering that overall more than 75% of endoscopic procedures leads to a positive outcome, the academic will seems to be lacking.^10^ Furthermore, trials comparing VAC therapy, clips, and simple SEMS repair in similar conditions could orient the future of this techniques and their relative indications. Finding a universal solution for every situation seems improbable, because the presentation and characteristics of the fistula determine the technique used after a somewhat practical approach that could, for example, be presented as an algorithm. For instance, larger fistulas are often treated with an approach combining VAC therapy and SEMSs as opposed to SEMSs or clips to treat smaller ones.

In conclusion, considering the association of low adverse events and high success rates with excellent technical outcomes, we consider endoscopic treatment as the most effective and safe first-line option for patients with small as well as large postoperative esophageal fistulas. Surgical intervention can stand in as an alternative in cases of repeated clinical failure or damage control.

## Disclosure

All authors disclosed no financial relationships.
